# Sexpertise: Sexual Knowledge and the Public in the Nineteenth and Twentieth Centuries

**DOI:** 10.1093/shm/hkae014

**Published:** 2024-04-03

**Authors:** Hannah Charnock, Sarah L Jones, Ben Mechen

**Affiliations:** University of Bristol, Bristol, UK; University of Bristol, Bristol, UK; University College London, London, UK

Histories of sexual knowledge are a well-established part of broader histories of sexuality but remain an exciting and productive part of the field. As Roy Porter and Mikulas Teich stated in their important 1994 collection *Sexual Knowledge, Sexual Science*, there has never been any consensus among historians about ‘how to tell the history of sexual knowledge and evaluate its ideological functioning in culture and society.’^1^ In the intervening 20 years, this lack of consensus has translated into some rich scholarship around the topic, as historians have done diverse work, using an array of sources and methodologies, to interrogate key questions around what sexual knowledge is, how it is acquired, where it circulates and who is able to speak on and through it.

In this introduction, we try to set out what we see as some of the key threads of this work, albeit without making claims—given both its brevity and the status of the authors as historians primarily of Anglo-America—to any kind of comprehensiveness. We also try to briefly position this special issue’s interest in ‘sexpertise’ in relation to this broader category of ‘sexual knowledge’, namely as knowledge about sex that travels, sometimes in unpredictable ways across media, across genres and between recognised sexual authorities, insurgent or apparently ‘illegitimate’ voices and a variety of publics.

Despite the multiplicity of viewpoints first highlighted by Porter and Teich, the continued and shared influence of Michel Foucault’s writings on the emergence of ‘sexuality’ as an object of scientific and medical knowledge has been notable. Indeed, in histories of sexual knowledge, explorations of sexology and *scientia sexualis* have remained hugely productive for historians looking to trace the ambivalent disciplinary formations underpinning or attempting to give a language to everyday sexual experience across the nineteenth and twentieth centuries.^2^

In a similar vein, studies in the history of psychoanalysis, with its foregrounding of the sexual substructures of personhood and pathology, have also in recent years been in rude health.

All the same, within this somewhat familiar matrix of interests, new areas of focus have emerged. To take two recent examples, books by Kirsten Leng and Michal Shapira have stressed the centrality of women to such early twentieth-century developments in sexual knowledge and claims to expertise, though now as agents and authorities rather than passive patients or the loosely drawn figures of masculine scientific imagination.^3^ Through such a lens, which has turned to the ideas of such marginalised figures as Johanna Elberskirchen (Leng) or Freud’s client Margarethe Csonka (Shapira), a history of sexual science that goes beyond such ‘fathers’ as Richard von Krafft-Ebing, Havelock Ellis or Sigmund Freud himself has become ever more apparent.

Indeed, as Leng and Katie Sutton have argued persuasively, at stake in much recent scholarship in the history of sexual knowledge has been just such a rethinking of the infamous boundary work and norm-setting of the sexual sciences and a related queering of their epistemological foundations.^4^ A further rich seam of studies, for example, has explored the development of sexology as a transnational, transimperial or global intellectual project, developing through innumerable moments of cross-cultural translation and dialogue, or else as one that grew along distinctive national or regional lines.^5^ Through this intellectual move of provincialisation, the strong association between sexology and Anglo-American or *Mitteleuropean* scientific traditions, arguably initiated by Foucault, has been steadily undone.

The environmental and posthuman turn, meanwhile, has encouraged a growing number of historians of sexuality to investigate sexology’s ambivalent traffic in ideas of ‘nature’ and the ‘natural’ or ‘man’ and (or as) animal. Such ideas could both undermine existing norms of sexual behaviour or identity, or else confirm them, often through the persuasive power of what the historian of science Lorraine Daston has called modernity’s ‘naturalistic fallacy’: the ‘smuggling’ of cultural values first into understandings of nature, and then the subsequent appeal to nature as the ground of such cultural values (whether they be sexually conservative by the standards of the time, or sexually radical).^6^ If sexual science, since Foucault, has occupied a privileged position in histories of the desiring human subject, this body of work has asked us to look again at the place of ‘nature’ in the convoluted definition of such a figure.^7^

Finally, there has been a growing recognition of what Heike Bauer has called the ‘porous’ edges of the field of sexology, and how its project has been consistently shaped by connections, overlaps and encounters between science, culture and politics in the broadest senses, and between sexology and adjacent areas of intellectual concern.^8^ Important research by Jana Funke and Aaron Stone, for example, has stressed the significance of literature as a site of such exchanges—in Stone’s terms, for example, through the development of a ‘black vernacular sexology’ that refused the racist suppositions of sexological orthodoxies.^9^

Following a related line of thought, other scholars have brought into question hierarchical or top-down models of how, where and by whom sexual knowledge is made, and then its transit across spaces of reading or consumption. Laura Doan, for example, has argued convincingly that historians need to move past didactic models of knowledge in which authorities of one kind or another simply bestow information upon lay publics, to instead recognise the more complex webs of interaction that have informed not only the communication of sexual knowledge but its creation, curation and adaptation.^10^ Peter Cryle and Elizabeth Stephens, in their pathbreaking ‘critical genealogy’ of normality, have shown how Alfred Kinsey’s aversion to ideas of a scientifically ‘normal’ sex was quickly overwritten by a popular interpretation of his surveys that saw an interest in the normal as their hallmark.^11^

Driven by such insights, fruitful work by scholars like Doan and Bauer, as well as Sarah Bull, H. G. Cocks and Roger Lancaster, has explored some of the intersections and encounters between science and popular culture, acknowledging their ‘two-way traffic’ in a way that has served to problematise previous visions of elite sexological knowledge and scientific expertise.^12^ Others have looked to investigate the transnational and cross-cultural connections that have shaped the construction of scientific sexual knowledge. Works such as Bauer’s *Sexology and Translation* as well as Fuechtner, Haynes, and Jones’ *Global History of Sexual Science*, for instance, have complicated assumptions about ‘experts’ and ‘the public,’ ‘science’ and ‘culture,’ by demonstrating how contested and contextually specific these notions have been in different cultural contexts.^13^ As Katie Sutton and Kirsten Leng have noted, work in this corner of the field is ‘expanding at a remarkable pace’ as historians of sexual science find helpful new ways to interrogate some of the murky ‘grey areas’ around what ‘counted’ as knowledge, the politics of who was said to have produced it, and what it meant to have scientific ‘expertise’ around sex in the past.^14^

As we have each argued elsewhere, one especially important place of interface between professional and lay understandings of sex in the modern period has been in sexual guidance literature.^15^ New histories in this area owe as much to the evolving social and cultural history traditions of the late-twentieth century as to intellectual and medical histories and this has translated into especially productive considerations of the form and audience of sexual knowledge.^16^ Though initially focussed on marriage guidance texts, as interest in the communication of sexual knowledge has moved to consider the mid and late-twentieth centuries, histories of sex advice have begun to consider magazines and periodicals, commercial literature and new media as key sites for sexual knowledge exchange and discussion of ‘the facts of life.’^17^ Reflecting the heteronormativity of ‘mainstream’ sexual culture in the twentieth century, the dilemmas and anxieties of ‘straight’ couples and individuals preoccupy these histories but this research has revealed the myriad struggles historic actors faced in embodying and achieving ‘normal’ sexuality and the varied strategies authorities proposed to guide them.^18^ Guidance of this sort could also question boundaries between sexology’s *scientia sexualis* and what Foucault counterposed as *ars erotica* (the erotic arts)—for example, by attempting to rest techniques for ‘pleasurable’ or ‘satisfying’ sex on some kind of scientific basis, a goal that had never especially troubled a figure like Krafft-Ebing. Such work has also again demonstrated that there has been no clear dividing line between ‘medical’ and ‘lay’ understandings of sex across the modern period and that, while expertise claimed by those offering advice and guidance was often medical or scientific in nature, such claims to sexual knowledge could take many forms and be communicated via, and decoded through, a range of media and languages.

Running in parallel to studies of sex guidance, scholars have also developed rich histories of sex education and the public communication of public health.^19^ This strand of research has carefully charted the different trajectories of sex education initiatives across the world in the modern era.^20^ Though the exact trajectories differ, reflecting the complex web of religious, social, political and cultural dynamics that informed such developments in different national, regional and (post-)colonial contexts, accounts of negotiation and debate have been central to these histories, reflecting the highly contested nature of sexual knowledge. In addition to showcasing the ways in which different authorities on sexual matters came into conflict, these histories have been particularly important in highlighting the ways in which matters of audience informed cultures of sexual knowledge exchange in the past. The dividing lines in debates over sex education have often been drawn not just over what types of sexual knowledge to communicate (moral, anatomical, medical, social and/or emotional) or over who has the authority to share sexual knowledge, but over who has the need or right to hear it.

In their heightened focus on the consumers of sexual knowledge, works on cultures of sex advice and histories of sex education push back against simplistic models in which expert-constructed knowledge is simply bestowed upon passive recipients. They have highlighted the ways in which sexual knowledge was constructed and adapted in order to speak to specific audiences and, at times, have been able to show how readers and consumers selectively adopted or resisted knowledge that did not map on to their own experience or speak to their needs or desires—or how they sought to develop counter-knowledge’s of sex through processes of critique, consciousness-raising and the celebration of the expertise of experience.^21^ As is often the case in audience studies, limited source material poses a consistent challenge to the researcher, however, and in many instances, it has not been possible to fully ascertain how historical actors understood or responded to attempts to advise, guide or educate them. Nevertheless, this work has been pivotal in showcasing the potential for thinking about sexual knowledge and expertise in terms of dialogue and/or exchange rather than expert-controlled didacticism.

The eight articles in this special issue all address different topics, themes and contexts, but together speak both to and across these important approaches to sexual knowledge in the past. Within individual articles and across the issue as a whole, contributors shed light on not only the ways in which institutional authorities such as the medical profession, the law, social science, religious organisations and businesses sought to construct and communicate information about sex in the nineteenth and the twentieth centuries but also consider claims to expertise made by individuals and communities, often on the basis of their own sexual lives. The central concern of this collection of articles is therefore to interrogate the messy interplays between ‘authorities,’ ‘experts’ and ‘publics’ in relation to sexual knowledge, and to think about connections, overlaps, and conversations between different strands of research in the field. By bringing these articles together, we look to explore how historical perspectives on definitions of sexual knowledge and explanations of how it functioned change when we draw from and reflect on these rich traditions in new and holistic ways.

To this end, we have found the label of ‘sexpertise’ a useful one for gathering together this special issue’s varied investigations of how sexual knowledge coalesces, becomes contested or travels. Despite the unserious or jokey origin of this term—it seems in mid-century newsstand coverage of figures like Alfred Kinsey and Helen Gurley Brown—we think it conveys well the conclusion that sexual knowledge has historically straddled realms of the ‘high’ and ‘low’, the elite and the popular, and accepted and unacceptable authorities.^22^

Bringing together a geographically and chronologically expansive series of articles by emerging and established scholars working in the UK, USA, Spain and Italy, the special issue reflects on ‘sexpertise’ as a complex field of practical, scientific, embodied and ethical knowledge about sexual practice that was under permanent (re)construction. Centrally, the special issue considers ‘sexpertise’ as a field of knowledge defined by its publicness. Put another way, articles in the special issue understand sexpertise as comprising claims about sex (as ‘good’ or ‘bad’, ‘safe’ or ‘unsafe’, ‘proper’ or ‘improper’, etc.) premised on a rejection of the notion that sexual behaviour belonged to a hallowed zone of personal privacy, patient–client confidentiality or rarefied understanding. Instead, authors here historicise ‘sexpertise’ as sexual knowledge that was shaped within, addressed to and legitimised by the various publics and counterpublics of nineteenth and twentieth-century mass societies.

To this end, the issue advances our understanding of a number of important themes. Articles by Tracey Loughran and Daisy Payling, by Ross Brooks, and by Francesca Campani, address the projection of sexual expertise by avowedly ‘popular’ texts, namely mass-market magazines and widely available sexological tracts, but also how readers responded to such ideas, becoming themselves active participants in this process. Mallory Szymanski and Mónica García-Fernández explore the shifting importance of religion in constructions of ‘sexpertise’, especially in the context of the apparent ‘secularisation’ of the public sphere, and examine the encounter between spiritual ideas about sex and new understandings of sexual selfhood. Elodie Serna considers how new, radical knowledge about sex and reproduction became central to the formation and political praxis of a transnational anarchist counterpublic. Finally, Katie Jones and Jen Grove think about how self-defined sexperts, from social scientists to historians of sexuality, construct their sexual publics as both objects *and* sources of sexual knowledge.^23^

Taken together, the articles demonstrate that whilst leading ‘sexperts’ were sometimes, as we might anticipate, writers of sex manuals or the spokespersons of prominent family planning associations, they were as often members of the transnational political underground, writers of clandestine literature, advertising copywriters or simply ‘ordinary’ people sharing sexual knowledge and experience within social and familial networks. As the special issue hopefully shows, only if ‘sexpertise’ is approached as an open, mutable and sometimes unruly category can the important place of publicly shared—or publicly contested—sexual knowledge in broader patterns of social and cultural change be fully registered.

## Footnotes

1 Roy Porter and Mikulas Teich, *Sexual Knowledge, Sexual Science: The History of Attitudes to Sexuality* (Cambridge: Cambridge University Press, 1994), 8.

2 Michel Foucault, *The History of Sexuality. Volume 1, The Will to Knowledge*, Robert Hurley (trans), (London: Allen Lane, 1979).

3 Kirsten Leng, *Sexual Politics and Feminist Science: Women Sexologists in Germany, 1900–1933* (Ithaca: Cornell University Press, 2018); Michal Shapira, *Sigmund Freud and His Patient Margarethe Csonka: A Case of Homosexuality in a Woman in Modern Vienna* (London: Routledge, 2023).

4 Kirsten Leng and Katie Sutton, ‘Histories of Sexology Today: Reimagining the Boundaries of *Scientia Sexualis*’, *History of the Human Sciences*, 2021, 34, 3–9.

5 A far from exhaustive list might include Katie Sutton, *Sex between Body and Mind: Psychoanalysis and Sexology in the German-speaking World, 1890s–1930s* (Ann Arbor: University of Michigan Press, 2019); Laurie Marhoefer, *Racism and the Making of Gay Rights: A Sexologist, His Student, and the Empire of Queer Love* (Toronto: University of Toronto Press, 2022); Dagmar Herzog, *Cold War Freud: Psychoanalysis in an Age of Catastrophes* (Cambridge: Cambridge University Press, 2016); Katerina Liskova, *Sexual Liberation, Socialist Style: Communist Czechoslovakia and the Science of Desire, 1945–1989* (Cambridge: Cambridge University Press, 2018); Chiara Beccalossi, *Female Sexual Inversion: Same-Sex Desires in Italian and British Sexology, c. 1870–1920* (Basingstoke: Palgrave Macmillan, 2012).

6 Lorraine Daston, ‘The Naturalistic Fallacy is Modern’, *Isis*, 2014, 105, 580.

7 For example, Kate Fisher and Jana Funke, ‘“Are We to Treat Human Nature as the Early Victorian Lady Treated Telegrams?”: British and German Sexual Science, Investigations of Nature, and the Fight against Censorship, ca. 1890–1940’, *Journal of the History of Sexuality*, 2024, 33, 79–101; Ina Linge, ‘The Potency of the Butterfly: The Reception of Richard B. Goldschmidt’s Animal Experiments in German Sexology around 1920’, *History of the Human Sciences*, 2021, 34, 40–70; Ross Brooks, ‘Darwin’s Closet: The Queer Sides of *The Descent of Man*’, *Zoological Journal of the Linnean Society*, 2021, 191, 323–346; Nadine Weidman, *Killer Instinct: The Popular Science of Human Nature in Twentieth-Century America* (Cambridge, MA: Harvard University Press, 2021); Erika Lorraine Milam, *Creatures of Cain: The Hunt for Human Nature in Cold War America* (Princeton: Princeton University Press, 2019). On a history of the subject as Foucault’s project, see Michel Foucault, ‘The Subject and Power’, *Critical Inquiry*, 1982, 8, 777.

8 Heike Bauer in Bauer and Ivan Crozier, ‘Sexology, Historiography, Citation, Embodiment: A Review and (Frank) Exchange’, *History of the Human Sciences* online (27 June 2017) [accessed 31 January 2024]. See also Kate Fisher and Jana Funke, ‘Sexual Science Beyond the Medical’, *The Lancet*, 2016, 387, 840–841; Benjamin Kahan, *The Book of Minor Perverts: Sexology, Etiology, and the Emergences of Sexuality* (Chicago: University of Chicago Press, 2019).

9 Jana Funke, ‘Lesbian-trans-feminist Modernism and Sexual Science: Irene Clyde and *Urania*, in R. Carroll and F. Tolan, eds, *The Routledge Companion to Literature and Feminism* (London: Routledge, 2023), 215–229; Aaron J. Stone, ‘Toward a Black Vernacular Sexology’, *GLQ: A Journal of Lesbian and Gay Studies*, 2023, 29, 27–42.

10 Laura Doan, ‘Troubling Popularisation: On the Gendered Circuits of a “Scientific” Knowledge of Sex’, *Gender & History*, 2019, 31, 304–318.

11 Peter Cryle and Elizabeth Stephens, *Normality: A Critical Genealogy* (Chicago: Chicago University Press, 2017), 346. See also Ruby Ray Daily, ‘“Dear Doctor K”: Mobility, Sex, and Selfhood in Alfred Kinsey’s British World Correspondence, 1948–58’, *Twentieth Century British History*, 2021, 32, 24–45.

12 Sarah Bull, ‘More Than a Case of Mistaken Identity: Adult Entertainment and the Making of Early Sexology’, *History of the Human Sciences*, 2021, 34, 10–39; Roger N. Lancaster, *The Trouble with Nature: Sex in Science and Popular Culture* (Berkeley: University of California Press, 2003); H. G. Cocks, ‘Saucy Stories: Pornography, Sexology, and the Making of Sexual Knowledge in Britain, c. 1918–70’, *Social History*, 2004, 29, 465–484.

13 Heike Bauer (ed.), *Sexology and Translation: Cultural and Scientific Encounters across the Modern World* (Philadelphia: Temple University Press, 2015); Veronika Fuechtner, Douglas E. Haynes and Ryan M. Jones (eds.), *A Global History of Sexual Science, 1880–1960* (Berkeley: University of California Press, 2017).

14 Leng and Sutton, ‘Histories of Sexology Today’, 5.

15 Hannah Charnock, ‘“How Far Should We Go?: Adolescent Sexual Activity and Understandings of the Sexual Life Cycle in Postwar Britain’, *Journal of the History of Sexuality*, 2023, 32, 245–268; Sarah L. Jones, ‘Science, Sexual Difference, and the Making of Modern Marriage in American Sex Advice, 1920–40’, *Gender & History*, 2023, 35, 249–266; Ben Mechen, ‘Alex Comfort’s *The Joy of Sex* and the Tensions of Liberal Sexpertise’, in T. Loughran, H. Froom, K. Mahoney and D. Payling, eds, *‘Everyday Health’, Embodiment, and Selfhood since 1950* (Manchester University Press, in press).

16 Alexander C. T. Geppert, ‘Divine Sex, Happy Marriage, Regenerated Nation: Marie Stopes’s Marital Manual *Married Love* and the Making of a Best-Seller, 1918–1955’, *Journal of the History of Sexuality* 1998, 8, 389–433.

17 A key starting point for this work was Roy Porter and Lesley H. Hall, *The Facts of Life: The Creation of Sexual Knowledge in Britain, 1650–1950* (New Haven: Yale University Press, 1995). See also Britta McEwen, ‘Emotional Expression and the Construction of Heterosexuality: Hugo Bettauer’s Viennese Advice Columns’, *Journal of the History of Sexuality* 2016, 25, 114–136; Hannah J. Elizabeth, ‘*Love Carefully* and Without “Over-bearing Fears”: The Persuasive Power of Authenticity in Late 1980s British AIDS Education Material for Adolescents’, *Social History of Medicine* 2021, 34, 1317–1342; Claire L. Jones, *The Business of Birth Control: Contraception and Commerce in Britain before the Sexual Revolution* (Manchester University Press, 2020); Daisy Payling, ‘Selling Shame: Feminine Hygiene Advertising and the Boundaries of Permissiveness in 1970s Britain’, *Gender & History*, 2023, 35, 1089–1110.

18 Jessamyn Neuhaus, ‘The Importance of Being Orgasmic: Sexuality, Gender, and Marital Sex Manuals in the United States, 1920–1963’, *Journal of the History of Sexuality*, 2000, 9, 447–473.

19 Some key examples include: Lutz D. H. Sauerteig and Roger Davidson (eds), *Shaping Sexual Knowledge: A Cultural History of Sex Education in Twentieth-Century Europe* (London: Routledge, 2009); Julian B. Carter, ‘Birds, Bees, and Venereal Disease: Toward an Intellectual History of Sex Education’, *Journal of the History of Sexuality*, 2001, 10, 213–249; Alison Bashford and Carolyn Strange, ‘Public Pedagogy: Sex Education and Mass Communication in the Mid-Twentieth Century’, *Journal of the History of Sexuality*, 2004, 13, 71–99; Virginie de Luca Barrusse and Anne Françoise Praz, ‘The Emergence of Sex Education: A Franco-Swiss Comparison, 1900–1930’, *Journal of the History of Sexuality*, 2015, 24, 46–74; Claudia Nelson and Michelle H. Martin (eds), *Sexual Pedagogies: Sex Education in Britain, Australia, and America, 1879–2000* (Basingstoke: Palgrave Macmillan, 2004); Agata Ignaciuk and Natalia Jarska, ‘Unawareness and Expertise: Acquiring Knowledge about Sexuality in Postwar Poland’, *Journal of the History of Sexuality*, 2023, 32, 121–143; Anne Hanley, ‘Migration, Racism and Sexual Health in Postwar Britain’, *History Workshop Journal*, 2022, 94, 202–222; Caroline Rusterholz, ‘Youth Sexuality, Responsibility, and the Opening of the Brook Advisory Centres in London and Birmingham in the 1960s’, *Journal of British Studies*, 2022, 61, 315–342; David Geiringer, *The Pope and the Pill: Sex, Catholicism and Women in Post-War England* (Manchester: Manchester Univesity Press, 2019).

20 Jonathan Zimmerman, *Too Hot to Handle: A Global History of Sex Education* (Princeton, NJ: Princeton University Press, 2015); Alexandra M. Lord, *Condom Nation: The US Government’s Sex Education Campaign from World War I to the Internet* (Baltimore, ML: Johns Hopkins University Press, 2010); Kristy L. Slominski, *Teaching Moral Sex: A History of Religion and Sex Education in the United States* (Oxford: Oxford University Press, 2021); Alessandra Aresu, ‘Sex Education in Modern and Contemporary China: Interrupted Debates across the Last Century’, *International Journal of Educational Development*, 2009, 29, 532–41; Jane Pilcher, ‘School Sex Education: Policy and Practice in England 1870 to 2000’, *Sex Education*, 2005, 5; Joy Talukdar, Tania Aspland and Poulomee Datta, ‘Sex Education in South Australia: The Past and the Present’, *Sex Education*, 2013, 13, 107–116.

21 Lucy Delap, ‘Rethinking Rapes: Men’s Sex Lives and Feminist Critiques’, *Contemporary British History*, 2022, 36, 253–276.

22 See, for example, ‘The Press’ [on Kinsey], *Newsweek* (8 May 1950), 60; Jane Howard, ‘Secretary’s Guide to Sex at High Noon’ [on Brown], *LIFE* (28 August 1964), 8.

23 Please see the Correction on page 719 for the three articles published in the previous two issues.

